# Association of Plasma Renalase and Left Ventricle Mass Index in Heart Failure Patients Stratified to the Category of the Ejection Fraction: A Pilot Study

**DOI:** 10.1155/2019/7265160

**Published:** 2019-10-14

**Authors:** Dijana Stojanovic, Valentina Mitic, Dejan Petrovic, Miodrag Stojanovic, Aleksandra Ignjatovic, Nikola Stefanovic, Tatjana Cvetkovic, Vladmila Bojanic, Gordana Kocic, Marina Deljanin Ilic

**Affiliations:** ^1^Institute of Pathophysiology, Faculty of Medicine, University of Nis, Dr. Zoran Djindjic Boulevard 81, 18000 Nis, Serbia; ^2^Institute for Treatment and Rehabilitation “Niska Banja”, Srpskih junaka 2, 18205 Niška Banja, Serbia; ^3^Department of Internal Medicine, Faculty of Medicine, University of Nis, Dr. Zoran Djindjic Boulevard 81, 18000 Nis, Serbia; ^4^Department of Medical Statistics and Informatics, Faculty of Medicine, University of Nis, Dr. Zoran Djindjic Boulevard 81, 18000 Nis, Serbia; ^5^Department of Pharmacy, Faculty of Medicine, University of Nis, Dr. Zoran Djindjic Boulevard 81, 18000 Nis, Serbia; ^6^Institute of Biochemistry, Faculty of Medicine, University of Nis, Dr. Zoran Djindjic Boulevard 81, 18000 Nis, Serbia

## Abstract

Heart failure represents a growing health problem, with increasing morbidity and mortality globally. According to the mechanisms involved in the pathogenesis of heart failure, many biomarkers have been proposed for the timely diagnosis and prognostication of patients with heart failure, but other than natriuretic peptides, none of them has gained enough clinical significance. Renalase, a new protein derived from kidneys was demonstrated to metabolize catecholamines and to have a cardioprotective role. The aim of the study was to determine whether renalase and brain natriuretic peptide (BNP) concentration could be used to differentiate heart failure patients stratified to the category of the ejection fraction and whether plasma renalase could be used as a biomarker for left ventricle hypertrophy in all subgroups of heart failure patients. We included patients diagnosed with heart failure and stratified them to the three subgroups according to the ejection fraction. Regarding echocardiographic parameters, HFmrEF had an intermediate profile in between HFrEF and HFpEF, with statistical significance in most evaluated parameters. BNP concentration was significantly different in all three subgroups (*p* < 0.001), and renalase was statistically higher in HFrEF (*p* = 0.007) compared to the HFmrEF and HFpEF, where its results were similar, without statistical significance. Renalase plasma concentration was demonstrated to be highly and positively associated with left ventricle mass index in HFrEF (*p* = 0.029), as well as increased plasma concentration of BNP (*p* = 0.006). In the HFmrEF group of patients, body mass index was positively associated with LVMI (*p* = 0.05), while in the patients with HFpEF, diabetes mellitus was demonstrated to have a positive association with LVMI (*p* = 0.043). These findings suggest that renalase concentration may be measured in order to differentiate patients with reduced ejection fraction. Plasma renalase concentrations positively correlated with left ventricle hypertrophy in patients with reduced ejection fraction, being strongly associated with increased left ventricular mass index.

## 1. Background

Heart failure (HF) represents a response to a previous cardiovascular injury presented with an abnormal cardiac structure or function, leading to increased intracardiac pressures or a decreased cardiac output [1], with increasing morbidity and mortality globally [2]. For the first time, the 2016 European Society of Cardiology (ESC) heart failure (HF) guidelines created three separate clinical entities, according to the left ventricle ejection fraction (LVEF), called heart failure with reduced EF (HFrEF), heart failure with mid-range ejection fraction (HFmrEF), and heart failure with preserved EF (HFpEF) [3].

In order to maintain cardiovascular and homeostasis in general, during the early stages of heart failure development, many physiological compensatory mechanisms are initiated, including the activation of the sympathetic nervous system (SNS) and cytokine secretion [4]. The activation of the SNS is able to preserve organ perfusion for a short time, but in the long term, it may lead to catecholamine-induced cardiotoxicity, resulting in reduced EF [4], while cytokine overproduction makes this a predictor of a worse outcome [5]. Therefore, the final result is ventricular remodeling, thus promoting heart failure progression [6].

In order to test the hypothesis that the kidney has an important role in preserving cardiovascular health, a new protein, subsequently called renalase, was discovered in 2005 [7], predominantly expressed in proximal tubules and to a lesser extent in the heart, liver, and skeletal muscles [7], after subtotal nephrectomy experiments in neonatal and adult rats showed that they develop left ventricular hypertrophy [7]. It was also demonstrated that soluble renalase degrades plasma catecholamines; therefore, it may have a significant role in blood pressure regulation [7–9]. Furthermore, two single nucleotide polymorphisms within the renalase gene were demonstrated to be risk factors for essential hypertension development [10]. Nevertheless, it was demonstrated that high plasma concentration of renalase may be a risk factor for the cardiovascular disease development and that it may predict all-cause mortality in patients with advanced kidney disease [11]. The most recent data, however, pointed to some other very important renalase roles, expanding its function beyond only being catalytic [12]. It was concluded that extracellular renalase may have cytokine properties [13], thus promoting cell survival.

Hypothesizing that renalase may reflect sympathetic activity, thus exerting cytokine properties, the aims of the study were to determine firstly whether renalase and brain natriuretic peptide (BNP) concentration could be used to differentiate heart failure patients stratified to the category of the ejection fraction and secondly whether plasma renalase could be used as a biomarker for left ventricle hypertrophy in all subgroups of heart failure patients.

## 2. Patients and Methods

The investigation was designed as a single-center, cross-sectional study and was conducted between May and October 2018 in the Institute for Treatment and Rehabilitation Niska Banja, in Nis, Serbia. All study participants were over 18 years old and gave written informed consent prior to the investigation. The study was approved by the institutional ethics committees. The study cohort represented 75 patients with a current history of HF, independent of etiology, and 35 healthy volunteers. All heart failure patients were clinically stable or in compensated heart failure status and had been admitted to the institute for the purpose of rehabilitation. The patients received the standard recommended pharmacological therapy [3]. The biochemical and clinical measurements were obtained within 24 hours of the hospital admission. The participants in the control group were community-based, without history of a coronary artery disease or heart failure and were age- and gender-matched with the participants in the clinical group.

The diagnosis of heart failure was established according to the presence of signs and symptoms of heart failure, BNP plasma concentration (over 35 pg/mL), relevant structural heart changes (LV mass index ≥ 115 g for males and ≥95 g for females or left atrial dilatation ≥ 40 mm), and/or diastolic abnormality (E/A ratio < 0.75 or ≥1.5 or deceleration time of E‐wave < 140 ms) [3].

Two-dimensional echocardiography was performed on all participants, using a commercially available system (Acuson Sequoia 256, New York) and was analyzed according to the current guidelines [14]. Left ventricular ejection fraction (LVEF) and LV volumes were obtained with biplane apical views (Simpson's biplane), and the dimensions of the left ventricle, left atrium, and LV mass were provided by M-mode imaging. The existence of mitral regurgitation was assessed systematically. Diastolic function was assessed by the E/A ratio, deceleration time, isovolumetric relaxation time, and E/E′ ratio. The structure and the function of the right heart were assessed by the dimensions of the right ventricle (RV), systolic pulmonary artery pressure, and tricuspid annular plane systolic excursion (TAPSE) in an apical 4-chamber view. The maximum systolic excursion of the lateral tricuspid annulus is measured by M-mode, with TAPSE of <17 mm indicating RV dysfunction [15]. Consequently, patients were divided into three subgroups according to the LVEF: HFrEF (LVEF < 40%, *n* = 27), HFmrEF (LVEF 40–49%, *n* = 23) and HFpEF (LVEF ≥ 50%, *n* = 25). Afterwards, their baseline characteristics and possible risk factors were compared.

All biochemical measurements were routinely obtained on the day of enrolment by using the apparatus Sysmex XS 1000, Europe GmbH. The serum concentrations of CRP were determined quantitatively, with a nephelometric test (Orion Diagnostica Turbox), and afterwards, plasma was stored and frozen at -80°C until all the samples were collected and prepared for biomarker quantification. Estimated glomerular filtration rate was assessed by the MDRD (Modification of Diet in Renal Disease) equation, while the subjects' most recent height and weight measurements were used to calculate body mass index.

The concentrations of evaluated biomarkers were measured in plasma samples using commercially available ELISA kits, according to the manufacturer's instructions. For renalase (USCN Life Science Inc., China) the range of detection was 3.12-200 ng/mL, and for BNP (Abcam, ab193694, United Kingdom), the minimum detectable dose was 14 pg/mL.

### 2.1. Statistical Analyses

Data are presented as mean ± standard deviation or as absolute and relative numbers. Data distribution was tested using a Shapiro-Wilk test, and normally distributed data were analyzed with one-way ANOVA and Tukey test. Nonparametric analysis of data was carried out with the Kruskal-Wallis and the Mann-Whitney test. Linear regression was used to establish the relationship between variables. The level of significance was set at *p* < 0.05, and all statistical analyses were performed using R software, version 3.0.3. (R Foundation for Statistical Computing, Vienna, Austria) [16].

## 3. Results

The total cohort of the study consisted of 110 participants, where 75 patients diagnosed with heart failure represented the clinical group and 35 community-based healthy volunteers were included as controls. The baseline characteristics of the study patients, stratified to the category of the ejection fraction, are presented in Table 1. Out of 75 patients, 27 (36.0%) were classified as patients with reduced heart failure (HFrEF), 23 patients (30.7%) had mid-range heart failure (HFmrEF), and 25 patients (33.3%) were classified as HFpEF. The mean age of the clinical group was 61.73 ± 10.02 (min 37, max 83 years), with no statistical significance between either the subgroups (*p* = 0.445) or the gender (*p* = 0.744). With regard to the etiology of the heart failure, coronary artery disease was most prevalent in the HFrEF patients (80.8%), compared to the HFmrEF (69.6%) and HFpEF (48%), with statistical significance between all three subgroups (*p* = 0.042). However, there were no significant differences between the subgroups regarding the other etiological factors for the development of heart failure. Analysis of the clinical history showed that hyperlipidemia was a statistically significant parameter in all three subgroups (*p* = 0.033), being the most prevalent in HFpEF patients and least in HFrEF. However, family history of cardiovascular disease was similarly prevalent in HFrEF and HFpEF patients, compared to HFmrEF patients (*p* = 0.033). Regarding biochemical measurements, there were statistically significant differences in blood urea nitrogen (BUN) concentration (*p* = 0.031), acidum uricum (*p* < 0.001), and triglycerides (*p* = 0.016) between all three subgroups, as shown in Table 2. The evaluated biomarkers, renalase and BNP, were statistically different compared to the control group (*p* < 0.001) (data not shown in the tables). BNP concentration was significantly different in all three subgroups (*p* < 0.001), and renalase was statistically higher in HFrEF (*p* = 0.007) compared to the HFmrEF and HFpEF where its concentrations were similar, without statistical significance (Table 2).

Regarding echocardiographic parameters, HFmrEF had an intermediate profile in between HFrEF and HFpEF, as shown in Table 3. Statistical significance was obtained using the following parameters: left ventricle ejection fraction (*p* < 0.001), left ventricle mass (*p* = 0.003), LV mass index (*p* = 0.005), LV end-systolic volume (*p* < 0.001), LV end-diastolic volume (*p* < 0.001), left atrium (*p* = 0.005), mitral regurgitation (*p* < 0.001), tricuspid regurgitation (*p* < 0.001), TAPSE (*p* < 0.001), and right ventricle (*p* = 0.003). However, the E/A and systolic pressure of RV were different between the subgroups, although this was not statistically significant.

**Figure 1:**
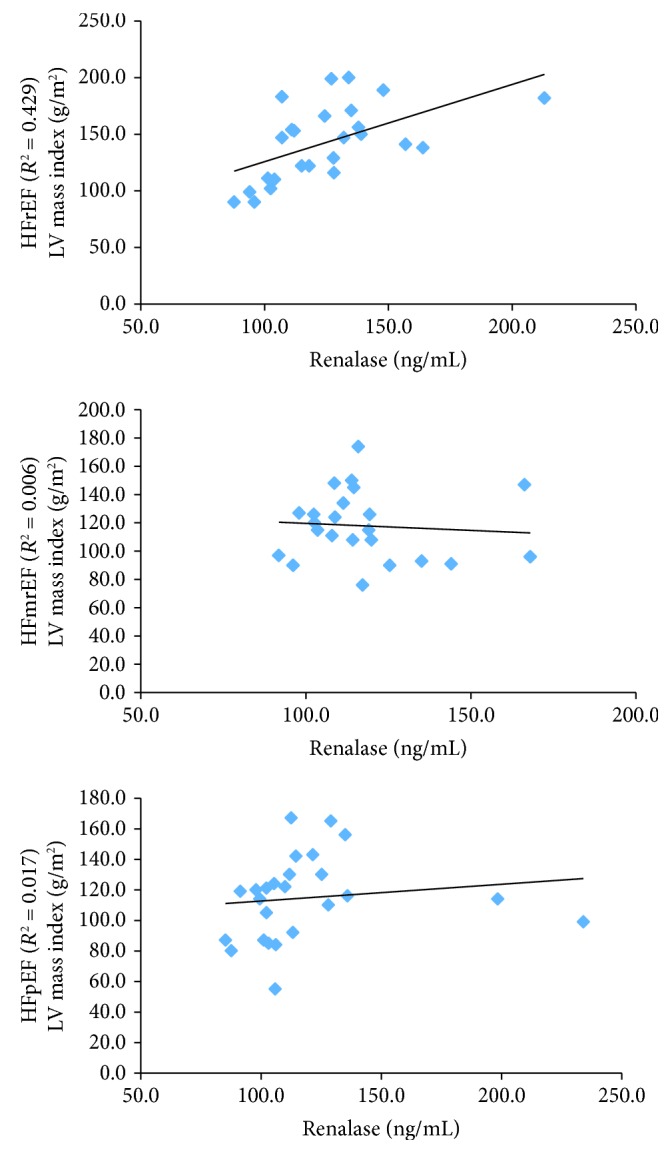
Association of renalase and left ventricle mass index stratified to the category of the ejection fraction. LV: left ventricle; HFrEF: heart failure with reduced ejection fraction; HFmrEF: heart failure with mid-range ejection fraction; HFpEF: heart failure with preserved ejection fraction.

Renalase plasma concentration was demonstrated to be highly and positively associated with the left ventricle mass index in HFrEF (*p* = 0.029), as well as increased plasma concentration of BNP (*p* = 0.006). In the HFmrEF group of patients, the body mass index was positively associated with LVMI (*p* = 0.05), while in the patients with HFpEF, diabetes mellitus was demonstrated to have a positive association with LVMI (*p* = 0.043), as shown in Table 4 and Figure 1.

## 4. Discussion

The first finding of the study is that plasma renalase concentration was higher in HFrEF patients, while HFmrEF and HFpEF patients showed similar results, which are lower levels of renalase. However, we also documented that soluble renalase exerted a very strong positive association with the left ventricle mass index, making it a significant risk factor for left ventricle hypertrophy. To the best of our knowledge, this is the first study to assess the potential role of plasma renalase in chronic heart failure patients, in the form of heart failure subtype differentiation and as a possible biomarker for left ventricle hypertrophy. The concentration of BNP was significantly different in all three subgroups of heart failure patients and was also demonstrated to have a strong and positive association with LVMI in patients with reduced ejection fraction.

As previously reported, sympathetic overactivation is one of the main mechanisms involved in the pathogenesis of heart failure, with catecholamines exerting direct toxic effects to the cardiomyocytes, by stimulation of apoptosis and induction of ventricular arrhythmias [17]. As noted, extracellular renalase plays a very important role in maintaining plasma catecholamine concentration, by metabolizing it [9]; therefore, it may be the key protein in the regulation of the sympathetic tone. However, it was concluded that soluble renalase firstly circulates as an inactive, in the form of a proenzyme, waiting for an influx of catecholamines to be activated and then *de novo* synthetized and secreted [9]. The causal relation between heightened sympathetic tone and increased cardiovascular risk was documented in patients with end-stage renal disease, who lack renalase; therefore, it was proposed that the application of recombinant renalase may exert a cardioprotective role [8–13, 17], while in patients with advanced chronic kidney disease, soluble renalase was proven to be a predictor of all-cause mortality [11].

Considering heart failure itself, in experimentally induced heart failure in rats, the activity and renalase concentration in plasma were both elevated compared with control animals [17]. Our heart failure patients also had higher plasma renalase levels compared to healthy controls, while the highest concentration of renalase was in the group of heart failure patients with the ejection fraction below 40%. We postulated that renalase concentration rises as a compensatory effect of the kidneys, as heart failure progresses, and is probably parallel to the activity of the sympathetic nervous system. Similar observations were documented in previous research [17], where renalase concentration was the highest during the first week of the experiment, corresponding to the early phase of heart failure development, and afterwards, as cardiac function declined, renalase returned to its base level. The authors proposed that while renal perfusion was preserved, renalase concentration tended to be elevated and *vice versa*, so the cardiac decompensation phase would be presented with subbasal renalase levels [17]. This finding is in agreement with our research. We discovered that the cohort with the reduced ejection fraction had the highest renalase levels, probably because they were all clinically compensated with a preserved kidney function. Our model indicated that renalase could be used as a differentiation biomarker in patients with ejection fraction below 40%. However, it could not be used, based on our findings, to differentiate heart failure patients with mid-range and preserved ejection fraction, since there was no statistical significance between plasma renalase concentrations in these two groups of patients. Impaired renal excretion also contributes to the rise of renalase concentration [18], but in our case, it is more likely that it reflects sympathetic activation [17], rather than the kidney dysfunction. However, further research is needed to decide the point at which renalase concentration starts to decline and the triggers of it.

Additional research has hypothesized that the increase in plasma renalase is due to its behaviour as a cytokine. However, some very important cytoprotective properties of renalase have been documented in the animal models of renal and cardiac injury [19]. In the experimental model of ischemic acute kidney injury, it was documented that after recombinant renalase was administered, the degree of kidney inflammation, apoptosis, and tubular necrosis was significantly reduced [20]. This protective effect of renalase against ischemia is probably provided by activation of renalase-dependent plasma membrane receptor, PMCA4b [21], with the final activation of mitogen-activated protein kinase (MAPK) and signal transducer and activator of transcription (STAT3) pathways [21]. Additionally, the same protective effect of renalase was observed in an animal model of ischemic myocardial damage [22]. The size and degree of an infarction area was attenuated after recombinant renalase was applicated. The administration of recombinant renalase was also demonstrated to prevent a significant fall in EF [22], after an ischemic injury. As reported, renalase may represent a target gene of hypoxia-induced factor-1 alpha (HIF-1*α*) [23, 24], but its protective effects may also be expressed through attenuating inflammation and reduction of necrosis and apoptosis [25]. The same authors proposed that renalase might have an important role in local heart tissue and different roles in different organs and that it might serve as a new therapeutic target for ischemic damage [25]. Furthermore, renalase may be involved in the pathogenesis of cardiac hypertrophy, most probably in the context of its prevention. In the rats, who underwent subtotal nephrectomy (5/6), after administration of renalase, the left ventricle hypertrophy, as well as left ventricle papillary muscle dysfunction, was significantly reduced [26]. Similarly, the development of hypertension and remodeling of the heart were attenuated most probably by inhibiting the profibrotic gene expression and phosphorylation of ERK-1/2 [19, 21, 27]. Our results may be analyzed in the context of renalase concentration being positively associated with left ventricle hypertrophy. We demonstrated that soluble renalase presents a risk factor for an increased left ventricular mass index, regardless of the type of hypertrophy. In an experimental model, it was confirmed that cardiac and renal fibrosis, as well as cardiac remodeling, was attenuated by exogenous renalase, suggesting that renalase may be involved in the pathogenesis of fibrosis, thus remodeling organs in some manner [26, 27].

Subsequently, a strong association between functional polymorphism of the renalase gene (Glu37Asp) and cardiac hypertrophy, ventricular dysfunction, poor exercise capacity, and ischemia was demonstrated in patients with coronary disease [28], further confirming a link between renalase, cardiac hypertrophy, and different aspects of myocardial function. The study confirmed that Asp/Asp homozygosity at codon 37 was connected with and increased left ventricular mass index [28], which is similar to our results where plasma renalase was confirmed to be a risk factor for an increase of LVMI (*p* = 0.029, *β* = 0.041) in heart failure patients with reduced EF. Note that our cohort of HFrEF patients consisted mostly of patients with coronary artery disease (over 80%), with ischemia being the main underlying mechanism. Therefore, we may presume that the elevated plasma renalase may be associated with the risk of coronary artery disease. Similar research was conducted, but the investigators reported the opposite that the decrease in plasma renalase was a significant risk factor for cardiovascular disease [29], explaining that the absence of renalase promotes myocardial cell apoptosis, oxidative stress, and fibrosis. The most recent genetic testing on the GG genotype of rs2576178 polymorphism concluded that this particular genotype increases renalase levels and thereby contributes to increased risk of coronary artery disease [30]. Some investigators recently also confirmed the association of renalase gene polymorphism with cardiac hypertrophy in female patients with aortic stenosis [31], again suggesting a causal relationship between renalase and cardiac hypertrophy. The authors postulated the potential link knowing that in hypoxia, glycolysis is increased and that renalase is secreted in the manner of preserving primary metabolism [31].

All the evidence suggests an important role for renalase in the development of cardiovascular diseases. According to the proposed mechanism, an excess of renalase probably occurs as a result of an adrenergic overstimulation, aiming to protect the tissues, with renalase being upregulated following different kinds of stress conditions [25, 26]. However, some investigators proposed that even a 10-fold increase in plasma renalase may not be sufficient to alleviate catecholamine levels [32]. Whether this elevation is of clinical significance reflecting patients who are at a higher risk of complications is yet to be confirmed. In this paper, we did not discuss the connection between plasma levels of renalase and BNP, but our results indicate that plasma renalase was a noninferior marker in the stratification of heart failure patients compared to plasma BNP, when discussing the correlation with left ventricle mass index. Monitoring of patients with heart failure may be facilitated by synergistically using plasma BNP and renalase, together with clinical parameters, in terms of diagnosis and determination of prognosis. Multimarker approaches have shown relevant benefit over single biomarkers, but additional research is needed to conclude the best biomarker combination for HF management [33].

## 5. Conclusion

In summary, plasma renalase concentration was higher in patients with heart failure than in the control group and was highest in patients with a reduced ejection fraction, compared to HFmrEF and HFpEF, where renalase concentration showed similar result. These findings suggest that plasma renalase levels may be measured in order to differentiate patients with ejection fraction below 40% serving as a potent biomarker for identification of heart failure patients with reduced ejection fraction. We also demonstrated that high plasma renalase concentrations positively correlate with left ventricle hypertrophy in patients with reduced ejection fraction, being strongly associated with increased left ventricular mass index.

The exact pathophysiological link between renalase and heart failure and its potential use as a biomarker in identification or risk stratification in heart failure patients is yet to be established.

## Figures and Tables

**Table 1:** Baseline demographic and clinical characteristics of heart failure patients stratified to the category of the ejection fraction.

Parameter	HFrEF *n* = 27		HFmrEF *n* = 23		HFpEF *n* = 25		*p* ^1^
Clinical							
Age (years)	59.44 ± 10.88		62.00 ± 9.54		63.96 ± 9.31		0.267^2^
Females (*n*, %)	6	22.2	7	30.4	6	24.0	0.791
HF etiology							
CAD (*n*, %)	21	80.8	16	69.6	12	48.0	0.042
CMP (*n*, %)	19	73.1	15	65.2	18	72.0	0.815
MI (*n*, %)	18	69.2	14	60.9	15	60.0	0.750
STEMI (*n*, %)	14	53.8	13	56.5	11	44.0	0.691
NSTEMI (*n*, %)	3	11.5	1	4.3	4	16.0	
VHD (*n*, %)	12	44.4	5	21.7	9	36.0	0.229
NYHA class (*n*, %)							
I	3	11.1	6	26.1	21	84.0	<0.001
II	7	25.9	16	69.6	4	16.0	
III	10	37.0	1	4.3	0	0.0	
IV	7	25.9	0	0.0	0	0.0	
Clinical history							
Hypertension (*n*, %)	24	88.9	23	100.0	22	88.0	0.099
Smoking (*n*, %)	14	51.9	10	43.5	12	48.0	0.840
Hyperlipidemia (*n*, %)	19	70.4	22	95.7	25	100.0	0.001
Family history (*n*, %)	19	70.4	9	39.1	18	72.0	0.033
Diabetes mellitus	7	25.9	7	30.4	10	40.0	0.546
BMI (kg/m^2^)	27.27 ± 4.22		29.58 ± 4.38		27.57 ± 3.48		0.126^2^
Obesity (*n*, %)	15	53.6	16	69.6	17	65.4	0.466
Anemia (*n*, %)	2	7.4	3	13.0	2	8.0	0.772
Depression (*n*, %)	5	18.5	2	8.7	4	16.0	0.582
Hemodynamics							
Systolic BP (mmHg)	122.22 ± 12.88		130.43 ± 14.21		127.60 ± 20.16		0.188^2^
Diastolic BP (mmHg)	75.74 ± 8.05		80.43 ± 8.78		78.40 ± 7.87		0.136^2^
MAP (mmHg)	91.22 ± 9.05		97.17 ± 9.50		94.72 ± 11.40		0.114^2^
PP (mmHg)	46.48 ± 8.86		50.00 ± 11.48		49.25 ± 14.63		0.480^3^

Continuous variables are expressed as mean ± standard deviation. ^1^Chi-squared test, ^2^ANOVA, and ^3^Kruskal-Wallis test. HFrEF: heart failure with reduced ejection fraction; HFmrEF: heart failure with mid-range ejection fraction; HFpEF: heart failure with preserved ejection fraction; HF: heart failure; CAD: coronary artery disease; CMP: cardiomyopathy; MI: myocardial infarction; STEMI: ST-elevation myocardial infarction; NSTEMI: non-ST segment elevation myocardial infarction; VHD: valvular heart disease; NYHA: New York Heart Association; BMI: body mass index; BP: blood pressure; MAP: mean arterial pressure; PP: pulse pressure.

**Table 2:** Baseline hematological and biochemical data with evaluated biomarkers of heart failure patients stratified to the category of the ejection fraction.

Parameter	HFrEF *n* = 27	HFmrEF *n* = 23	HFpEF *n* = 25	*p* ^1^
RBC (10^12^/L)	4.76 ± 0.46	4.85 ± 0.59	4.70 ± 0.52	0.613^2^
WBC (10^9^/L)	7.42 ± 1.82	8.14 ± 1.96	12.83 ± 27.39	0.445
Platelets (10^3^/mm^3^)	209.15 ± 57.85	257.83 ± 70.70	217.25 ± 76.84	0.137
Hemoglobin (g/L)	142.18 ± 14.36	141.96 ± 14.34	136.72 ± 11.44	0.272^2^
Hematocrit (%)	0.42 ± 0.04	0.42 ± 0.04	0.41 ± 0.03	0.418^2^
C-reactive protein (mg/L)	2.22 ± 5.81	0.52 ± 2.50	0.48 ± 2.40	0.259
ESR	17.13 ± 12.05	19.56 ± 12.86	18.56 ± 9.04	0.622
Creatinine (*μ*mol/L)	109.73 ± 25.42	100.54 ± 20.26	99.78 ± 24.90	0.279
BUN (mmol/L)	8.69 ± 4.34^a,b^	6.61 ± 2.65	6.27 ± 1.65	0.031
Acidum uricum (mmol/L)	455.93 ± 129.26	395.26 ± 66.97	320.32 ± 91.23	<0.002
Glycemia (mmol/L)	6.37 ± 1.91	6.72 ± 2.01	6.59 ± 2.12	0.652
HbA1c (%)	7.70 ± 1.72	7.62 ± 2.62	9.24 ± 1.14	0.234
TC (mmol/L)	4.86 ± 1.14	4.92 ± 1.49	4.49 ± 1.45	0.321
Triglycerides (mmol/L)	1.61 ± 0.57	1.97 ± 0.79	1.46 ± 0.77	0.016
LDL (mmol/L)	3.05 ± 0.97	3.06 ± 1.32	2.86 ± 1.15	0.603
HDL (mmol/L)	1.09 ± 0.29	0.97 ± 0.13	1.02 ± 0.24	0.666
LDL/HDL	2.84 ± 0.81	3.10 ± 0.97	2.83 ± 0.99	0.533
TC/HDL	4.56 ± 0.93	5.04 ± 1.06	4.43 ± 1.22	0.147
TG/HDL	1.70 ± 0.87	2.02 ± 0.87	1.47 ± 0.85	0.050
eGFR (mL/min/1.73 m^2^)	65.74 ± 22.84	76.09 ± 23.79	77.84 ± 22.54	0.148
Renalase (ng/mL)	147.33 ± 29.07^a,b^	118.58 ± 19.61	117.31 ± 32.83	0.007
BNP (pg/mL)	276.12 ± 200.73^a,b^	152.46 ± 27.53^c^	93.19 ± 19.31	<0.001

Continuous variables are expressed as mean ± standard deviation. ^1^Kruskal-Wallis test, ^2^ANOVA, ^a^HFrEF vs. HFmrEF, ^b^HFrEF vs. HFpEF, and ^c^HFrEF vs. HFpEF. RBC: red blood cells; WBC: white blood cells; CRP: C-reactive protein; ESR: erythrocyte sedimentation rate; BUN: blood urea nitrogen; TC: total cholesterol; TG: triglycerides; LDL: low-density lipoprotein; LDL: low-density lipoprotein; HDL: high-density lipoprotein; eGFR: estimated glomerular filtration rate; BNP: brain natriuretic peptide.

**Table 3:** Echocardiographic parameters of heart failure patients stratified to the category of the ejection fraction.

Parameter	HFrEF *n* = 27	HFmrEF *n* = 23	HFpEF *n* = 25	*p* ^1^
LVEF (%)	26.30 ± 5.98^a,b^	41.43 ± 2.25^c^	53.84 ± 3.64	<0.001
LV mass (g)	288.00 ± 79.18^a^	230.70 ± 43.98^c^	229.00 ± 51.11	0.003
LV mass index (g/m^2^)	147.52 ± 44.15^a,b^	117.87 ± 24.32	114.68 ± 27.55	0.005
LV end-systolic volume (mm)	48.28 ± 8.35^a,b^	36.78 ± 2.94	35.40 ± 3.08	<0.001
LV end-diastolic volume (mm)	63.70 ± 5.98^a,b^	55.65 ± 5.05	52.04 ± 4.57	<0.001
IV septum (mm)	10.87 ± 1.28	11.2 ± 1.27	11.59 ± 1.67	0.161
LV posterior wall (mm)	9.89 ± 1.24	9.93 ± 0.97	10.19 ± 1.14	0.539
Left atrium (mm)	47.02 ± 4.32^a,b^	44.26 ± 4.88	42.40 ± 5.90	0.001
Aortic root (mm)	34.54 ± 3.4	34.63 ± 4.1	34.51 ± 4.36	0.994
AR	0.41 ± 0.75	0.22 ± 0.52	0.23 ± 0.55	0.441
MR	1.93 ± 0.96^a,b^	1.48 ± 0.73^c^	0.91 ± 0.89	<0.001
TR	1.48 ± 0.70^b^	1.35 ± 0.65^c^	0.94 ± 0.68	0.006
TAPSE (mm)	18.39 ± 2.35^a,b^	21.83 ± 4.1^c^	24.23 ± 4.38	<0.001
Right ventricle (mm)	24.22 ± 2.74^1,2^	22.42 ± 2.59	22.32 ± 2.08	0.003
Systolic pressure of RV (mmHg)	36.96 ± 11.90	32.30 ± 8.92	31.56 ± 10.51	0.124
E/A	0.87 ± 0.27	0.83 ± 0.19	0.76 ± 0.14	0.442
LVMI > RF	22 (81.5)	13 (56.5)	14 (56.0)	0.089^2^

Continuous variables are expressed as mean ± standard deviation. ^1^Kruskal-Wallis test, ^2^chi-squared test, ^a^HFrEF vs. HFmrEF, ^b^HFrEF vs. HFpEF, and ^c^HFmrEF vs. HFpEF. HFrEF: heart failure with reduced ejection fraction; HFmrEF: heart failure with mid-range ejection fraction; HFpEF: heart failure with preserved ejection fraction; LVEF: left ventricle ejection fraction; LV: left ventricle; IV: interventricular septum; AR: aortic regurgitation; MR: mitral regurgitation; TR: tricuspid regurgitation; TAPSE: tricuspid annular plane systolic excursion; RV: right ventricle; LVMI > RF: LVMI above reference value.

**Table 4:** Association of evaluated parameters and LVMI in heart failure patients stratified to the category of the ejection fraction.

	Unstandardized coefficient		Standardized coefficient	
	B	Std. error	*β*	*p*
HFrEF				
Age	0.14	0.66	0.0	0.86
Gender	-1.21	14.07	-0.01	0.92
Renalase (ng/mL)	0.6	0.27	0.41	0.029
BNP (pg/mL)	0.11	0.04	0.52	0.006
Diabetes mellitus	-4.2	.5	-0.18	0.222
BMI (kg/m^2^)	-1.6	1.	-0.1	0.20
Constant	94.28	6.47		0.011
Adjusted *R*^2^	0.657			<0.001
HFmrEF				
Age	-0.67	0.65	-0.26	0.19
Gender	-11.8	14.8	-0.2	0.47
Renalase (ng/mL)	0.02	0.5	0.02	0.957
BNP (pg/mL)	0.25	0.26	0.28	0.42
Diabetes mellitus	0.6	2.84	0.05	0.828
BMI (kg/m^2^)	-2.5	1.2	-0.45	0.05
Constant	204.18	59.82		0.004
Adjusted *R*^2^	0.016			0.425
HFpEF				
Age	0.7	0.52	0.25	0.175
Gender	-11.	11.19	-0.18	0.24
Renalase (ng/mL)	0.18	0.15	0.21	0.28
BNP (pg/mL)	0.15	0.26	0.10	0.576
Diabetes mellitus	-5.24	2.40	-0.40	0.04
BMI (kg/m^2^)	-2.44	1.7	-0.1	0.092
Constant	149.4	64.45		0.02
Adjusted *R*^2^	0.66			0.02

LVMI: left ventricle mass index; HFrEF: heart failure with reduced ejection fraction; HFmrEF: heart failure with mid-range ejection fraction; HFpEF: heart failure with preserved ejection fraction; BNP: brain natriuretic peptide; BMI: body mass index.

## Data Availability

The data.xls used to support the findings of this study are available from the corresponding author upon request.
